# Pain Retrained: Participant Perspectives of an Online, Interdisciplinary Chronic Pain Education Programme

**DOI:** 10.1002/ejp.70223

**Published:** 2026-01-29

**Authors:** Maura McCarron, Francis Agnew, Claire Briggs, Jason Brooks, Martin Dempster, Jackie Granleese, Danielle Rainey, Kevin E. Vowles

**Affiliations:** ^1^ School of Psychology Queen's University Belfast Belfast UK; ^2^ Belfast Centre for Pain Rehabilitation Belfast Health and Social Care Trust Belfast UK; ^3^ Psychological Services Research and Development Centre Belfast Health and Social Care Trust Belfast UK; ^4^ The Research and Development Division of the Northern Ireland Health and Social Care Service Belfast UK

**Keywords:** chronic pain, interdisciplinary, online education, qualitative research, videoconference

## Abstract

**Background:**

Education is considered a foundational component of chronic pain treatment. In response to challenges relating to accessibility and scalability, health services have increasingly adopted online formats for delivering pain education programmes. However, little is known about how patients experience these digital interventions, particularly in relation to engagement, meaning‐making, and perceived impact. This study aimed to explore participant experiences of completing an online education programme for chronic pain.

**Methods:**

The study adopted an Interpretative Phenomenological Analysis (IPA) methodological framework, which informed the development of the interview schedule, the sampling strategy, and the analytic process. Semi‐structured interviews were conducted with 10 adults who completed a 6‐week programme, Pain Retrained, which delivered education on chronic pain in a group setting via Microsoft Teams. The intervention was provided by an interdisciplinary team with 2 h of clinician contact per week.

**Results:**

Analyses identified two principal themes, “It was a stretch” and “And now I can move on”. Participants expanded their understanding of chronic pain from a biomedical model to a more integrated biopsychosocial perspective, which supported a constructive shift toward engaging with evidence‐based care and self‐management.

**Conclusion:**

This study offers novel insights into participants' experiences of an online pain education programme. The online format enhanced accessibility and made a longer‐duration intervention more feasible for individuals. This structure allowed relational and temporal processes to support reconceptualization and importantly, reorientation to evidence‐based care. Findings highlight the value of participatory, interdisciplinary education delivered in a format that accommodates the realities of living with chronic pain.

**Significance Statement:**

This research advances understanding of how education can lay the foundation for recovery in chronic pain. A pivotal contribution is the identification of *reorientation to evidence‐based treatments* as a core process within pain education. These findings extend current models of pain education by highlighting experiential elements critical to change. The insights offer actionable direction for clinicians and service leadership seeking to develop, implement or refine accessible pain education interventions.

## 1 Introduction

Chronic pain, defined as pain persisting for at least 3 months (Treede et al. 2015), represents a major public health challenge (Reed et al. 2025), affecting approximately 28% of the population worldwide (Zimmer et al. 2022). It frequently disrupts multiple domains of daily life, including physical functioning, mood, and relationships, significantly diminishing individuals' ability to participate in meaningful activities (Duenas et al. 2016; Fine 2011).

Treatment remains clinically complex due to its biopsychosocial nature (Souza et al. 2025; Walumbe and Denneny 2025). Best practice typically involves interdisciplinary care, with patient education recognised as a foundational component (Fisher and De C Williams 2025; Mardon et al. 2024) and clinical guidelines consistently recommending pain education as a core treatment element (Macfarlane et al. 2017). However, despite its prominence in policy, access to education, particularly that delivered by interdisciplinary teams, remains limited and is frequently deprioritised (G. L. Moseley 2019). Even where such education is available, patient access may be hindered by barriers including mobility issues and travel challenges. For people with chronic pain, these factors can make in‐person attendance burdensome, limiting engagement even when services are offered (Slattery et al. 2019).

To address these challenges, many services have adopted online delivery formats, which offer the potential to improve access to pain education (Tauben et al. 2020). One approach is videoconferencing, which maintains the possibility of real‐time interaction with clinicians without the need for travel. Online education may thus offer a more equitable and scalable pathway to delivering guideline‐recommended care (Heapy et al. 2015), however, concerns remain, particularly around difficulties in establishing therapeutic connection in online settings (Eccleston et al. 2020). Crucially, it cannot be assumed that online interventions are experienced in the same way as those delivered face‐to‐face. Despite the expansion of online education, there is limited qualitative research exploring how people living with chronic pain experience these interventions. While qualitative studies have explored telehealth more broadly in chronic pain care (Fernandes et al. 2022), most evaluations of education interventions rely on quantitative outcome measures which risk overlooking the nuanced, subjective dimensions of patient experience (Buhrman et al. 2016). This includes how individuals engage with the intervention and integrate or resist its messages in daily life. Moreover, patient views are rarely included in the development or evaluation of pain education interventions, whether in‐person or online (Ernstzen et al. 2022; Leake et al. 2021). Experiential accounts can highlight barriers and facilitators to engagement and thus directly inform the refinement of interventions to better meet patients' needs.

Qualitative methods, particularly Interpretative Phenomenological Analysis (IPA), offer a way to address this gap by exploring how individuals experience, interpret, and find meaning in their participation (Smith et al. 2009). This study aims to explore the lived experiences of individuals who participated in a novel online, interdisciplinary, group pain education programme—Pain Retrained. By attending to the complexities of patient experience, this research seeks to inform the development and implementation of pain education interventions which are grounded in the needs and perspectives of those they are intended to support.

## 2 Method

### 2.1 Study Design

This study employed a qualitative, cross‐sectional design using semi‐structured interviews to explore how individuals experience and make sense of the Pain Retrained programme. Interpretative phenomenological analysis (IPA) was selected as the most appropriate methodological approach given the study's aim. These kinds of analyses are grounded theoretically in phenomenology, hermeneutics and idiography and enable an in‐depth examination of how individuals interpret and give meaning to significant personal experiences (Smith 2011; Smith et al. 2009).

A patient and public involvement (PPI) partner was involved in developing the interview schedule and participant facing documents and is a co‐author on this paper. This study was approved by the United Kingdom's National Health Service (NHS) Health Research Authority East Midlands‐Derby Research Ethics Committee (REC reference: 24/EM/0027) and was pre‐registered on the Open Science Framework (https://doi.org/10.17605/OSF.IO/VXPDB). It is reported in line with the Consolidated Criteria for Reporting Qualitative Studies (COREQ) framework (Tong et al. 2007).

### 2.2 Intervention

The Pain Retrained programme was developed and delivered by the clinical team in a Northern Ireland‐based secondary care pain service within the NHS. It comprised six weekly online education sessions, each lasting 2 h, delivered synchronously via Microsoft Teams. Content was informed by the biopsychosocial model and cognitive‐behavioural approaches to chronic pain. In brief, sessions included both didactic and interactive elements and focused on topics such as current scientific understanding of chronic pain as a biopsychosocial phenomenon, and the role of activity, movement, sleep, stress, and medication. The programme was facilitated by an interdisciplinary team which consisted of a medical doctor, clinical psychologist, physiotherapist, occupational therapist, and specialist pain nurses. The intervention has been more comprehensively described elsewhere (McCarron et al., under review) and details are provided in Supporting Information (Appendix S1). In the present study, individuals were recruited from Pain Retrained cohorts conducted over a seven‐month period. Group sizes ranged from 15 to 20 and each cohort received the same standardised programme content.

The Pain Retrained programme constitutes the first point of contact for most patients referred into this secondary care pain service. Previous work characterising this patient population indicates that individuals referred to the service typically present with complex, widespread chronic pain with an average pain duration of 12.3 years (SD: 10.1; Vowles et al. 2025).

### 2.3 Participants

Eligibility for the present study required completion of Pain Retrained. During the penultimate and final sessions of each 6‐week programme, the clinical team verbally introduced the research study to the entire patient group and subsequently distributed the participant information sheet via email. Interested individuals contacted the lead researcher (MM) directly. A purposive sampling strategy, consistent with IPA methodology, was used and recruitment continued until the target sample size of 10 was reached, consistent with recommendations for IPA studies (Smith et al. 2009). Participants also had the opportunity to ask questions of the research team (MM, KEV, MD). Prospective participants were informed the research was being carried out in part fulfilment of the lead researcher's PhD. All researchers were previously unknown to the participants. All participants provided written informed consent via secure electronic means prior to taking part. As detailed in the study information sheet, there was no financial compensation for participation. Data collection took place from September 2024 to April 2025. The sample included six females and four males, aged 42 to 68 years (median = 57 years). Pain duration ranged 4 to 25 years (median = 13 years). Individual participant characteristics are shown in Table S1.

### 2.4 Data Collection

Semi‐structured interviews were used in keeping with IPA methodology to facilitate openness and give participants the opportunity to tell their own story (Smith et al. 2009). The interview schedule (see Appendix S2 in the Supporting Information) was designed to explore the participants' experience of undertaking Pain Retrained via open‐ended, non‐leading questions and prompts (Smith et al. 2009; Smith and Nizza 2022). Participants were given the opportunity to lead the interview and make sense of their experiences through highlighting issues important to them, while simultaneously permitting the researcher freedom to probe further on topics related to the aims of the study (Smith 2011). Interviews were conducted and recorded by the lead researcher on Microsoft Teams and ranged in duration from 45 to 60 min. No‐one other than the participant and the researcher contributed to the interviews. The interviewer introduced herself as a PhD student and reassured participants that their responses would be confidential and have no impact on their clinical care. Each interview was followed by a debrief and no‐one requested further support. Participants also completed a brief demographic and pain history questionnaire at the beginning of the interview to contextualise the data. Recordings were transcribed verbatim using a combination of Microsoft Teams automated transcription and manual checking by the lead researcher. Transcripts were then pseudonymized and recordings deleted.

### 2.5 Data Analysis

#### 2.5.1 Analysis Procedure

Interview data were analysed within the IPA framework which focuses on the participant's subjective experience, and is interpretative in nature, acknowledging the active role of the researcher in making sense of those experiences. Meaning is co‐constructed and shaped by both the participant's account and the researcher's interpretative engagement, influenced by their respective backgrounds, assumptions, and experiences (Smith et al. 2009; Smith and Nizza 2022).

Initial analysis was conducted by one researcher (MM) and followed the detailed procedure and updated terminology outlined by Smith and Nizza (2022). An inductive, idiographic approach was adopted throughout the first stage of analysis. Each transcript was analysed independently and in full, allowing for a deep engagement with the individual's narrative and space for reflexive interpretation. The researcher began by reading and re‐reading each transcript to develop familiarity. Exploratory notes were made during subsequent readings of each transcript. Microsoft Word was used to facilitate the analysis, with analytic comments recorded in adjacent columns alongside the transcript text. The researcher attended to descriptive, linguistic, and conceptual content, highlighting passages that appeared particularly salient or resonant. A line‐by‐line analysis was undertaken, during which the researcher noted personal reflections. From these exploratory notes, experiential statements were developed, marking a shift toward abstraction and interpretation (Smith and Nizza 2022). These statements, recorded in a separate column alongside the transcript, represented the researcher's effort to capture the essence of the participant's lived experience. The next phase involved identifying personal experiential themes by clustering related experiential statements, thus providing a coherent summary of each participant's meaning‐making process.

Following the completion of idiographic analyses for each individual transcript, a cross‐case analysis was conducted to explore patterns of convergence and divergence across personal experiential themes. Group experiential themes were developed by clustering together themes that demonstrated conceptual similarity or resonance across participants' accounts. The process involved iterative refinement to ensure themes remained grounded in the data, reflecting both the shared and unique aspects of participants' experiences. Themes were named and defined to capture their conceptual essence while preserving their connection to the original narratives. The developing thematic structure was discussed in depth and further refined collaboratively by all members of the research team. Throughout the process, the analysis was recursive and reflexive, involving constant movement between the data and the developing themes. Themes are presented later in this report.

#### 2.5.2 Analysis Evaluation

To strengthen the quality and rigour of the study, the research team adhered to guidelines and principles specific to IPA, following criteria outlined by Smith (2011), Nizza et al. (2021) and Yardley (2000). All stages of the analysis focused on understanding each participant's individual experience in depth. The researchers carefully reviewed the transcripts, paid attention to participants' narratives, and considered both similarities and differences across accounts.

In line with Smith's (2011) position, respondent validation was not conducted, as the interpretative nature of IPA involves abstractions that may not be accessible to participants and could be influenced by perceived power imbalances.

#### 2.5.3 Reflexivity and Positionality Statement

In line with IPA's interpretative and idiographic commitments, the researchers explicitly acknowledge their influence on the research process (Cena et al. 2024; Olmos‐Vega et al. 2022; Yardley 2000). The lead researcher is a white, female rheumatologist with a professional interest in chronic pain and personal experience of chronic pain through family members. These factors positioned her as a partial insider, with contextual understanding of the phenomenon, while her lack of direct lived experience positioned her as an outsider in other respects. This dual insider–outsider position enabled both empathic engagement and critical distance in interpretation. A reflexive journal and detailed audit trail were maintained throughout the research process to document assumptions and analytic decisions. Regular supervision and debriefing with senior team members experienced in chronic pain treatment and qualitative methods, including IPA, helped to support reflexivity and enhance the transparency, credibility, and rigour of the interpretative process.

## 3 Results

Participants' experiences were interpreted and conceptualised within two Group Experiential Themes: (1) “It was a stretch” and (2) *“*And now I can move on”. These themes and related subthemes are described below and illustrated by direct quotations. Pseudonyms are used in place of participants' names to maintain confidentiality. Overall interpretation of the data suggests that while participants initially found the online group education programme challenging, it ultimately provided a valuable framework for reorienting toward new approaches to understanding and managing chronic pain.

### 3.1 (1) “It Was a Stretch”

This theme encapsulates participants' accounts of initial discomfort and challenge in engaging with the programme, alongside their progressive recognition of its benefits. The idea of being “stretched” operated on multiple levels—emotionally, cognitively, and physically, reflecting both the difficulty of re‐evaluating long‐held beliefs and the expansion of personal capability that emerged through participation.

#### 3.1.1 Challenge and Growth

The challenges and growth experienced during the programme were captured in participants' repeated use of the word “stretched,” which appeared in multiple, varied contexts and gave rise to the theme of stretching drawn directly from their language. Susan expressed how she was initially concerned about the online format. “At the start I was definitely scared about it, it was a real stretch for me, you know, having to do it on the computer and what if I made a mistake?” however she then continues, “But actually I was really pleased I got it to work and it's actually given me a little boost you know, like I'm a computer person now! My grandkids think it's funny”. She moves from a position of fear and self‐doubt to agency and self‐expansion to include the new identity of “computer person”. Her repetition of the word “actually” seems to convey her surprise and delight in this occurrence.

Multiple participants described feeling confronted by some of the programme content, particularly in learning that medication was not the primary solution and that their past treatments and behaviours may have been counterproductive, as vividly captured by James. “I just felt like I've been doing the wrong things all these years, why on earth did nobody tell me this before? Here I was thinking I'm just so angry, although better late than never I suppose”. This account conveys both a sense of betrayal and belated relief, capturing the emotional complexity of learning something valuable that might have altered his trajectory if received earlier. It reflects a moment of cognitive dissonance and the beginning of a reflective shift. The discomfort of re‐evaluating personal health strategies was central to the experience of “stretching” as he goes on to say, “at least now I know there are other things to try and we're all in the same boat”. Importantly, as conveyed by use of the metaphor “all in the same boat”, the group format seemed to create a space in which participants could process these challenges collectively, mitigating the potential inherent shame. Anne comments, “I was just so glad I wasn't the only one who seemed to think painkillers were the be‐all and end‐all”. The use of the colloquialism here very eloquently conveys the general misconception that so many felt or hoped that medication could be the solution.

Participants began to reframe their pain through a broader lens, one that included psychological and social dimensions. Anne reflects, “I think back to how I used to be so confused when my GP kept suggesting counselling, I mean, like I thought the pain is in my back surely I need to see a spinal surgeon.” This reflection suggests she is moving toward a more biopsychosocial conceptualisation of chronic pain, her use of the past tense implying she is no longer quite so rooted in the biomedical model. Conversely, James struggled with this shift, “It seemed a bit of a stretch to me that something like going for coffee with a friend could help in terms of my pain,” capturing the scepticism many of the participants seemed to feel, particularly in the early stages of the programme. Interestingly, accounts of “stretching” were also described in the physical context, as Margaret explains.

Before, I always thought resting was the safe thing to do when my pain was bad. Now I know that moving, even just stretching is what's recommended. So now I do a big stretch every morning, just like my dog!

Learning about the role of physical movement and gradual exposure to activity offered a tangible, embodied counterpart to the cognitive and emotional shifts taking place. With the reference to her pet, there is the impression that this participant is reframing and connecting with the value of movement as something natural to do.

#### 3.1.2 Ten Teachers in My Living Room

Extending the subtheme of Challenge and Growth, participants identified a range of opportunities associated with the online group format of the education programme, beginning with its accessibility and convenience. Frank stated, “My mobility's just not very good”, reflecting the straightforward yet significant impact of physical limitations. Lisa explained, “If I'd had to come into the hospital for this, I probably would have missed over half the sessions, there's just so many days I'm not up to leaving the house”. The phrase “I'm not up to it” was echoed across multiple interviews, with participants offering similar reflections, such as Susan's comment, “I can't face going out” and Mark noting, “I couldn't do it if it wasn't online”. These statements pointed not only to physical barriers but also to emotional and psychological ones, suggesting that for some, in‐person attendance would have felt overwhelming or unmanageable. Together, these accounts highlight how the online delivery format enabled participation by reducing both logistical and emotional barriers, allowing participants to access the programme on their own terms and in a way that felt possible, even on more difficult days.

Several of the participants referred to appreciating the fact they were able to engage with the programme from the safety and privacy of their own home. Lisa began, “I'm a background person you know, one of those background people. I tend to sit in the background. But that was ok here”. The repetition of “background” conveys her self‐identification as someone who typically remains on the peripheries of group interaction, giving the impression of withdrawal or perhaps marginalisation. The subsequent qualifying phrase “But that was ok here” introduces a subtle shift, a sense that she felt accepted and that her limited participation was not perceived as a deficit. She depicted an environment rich with potential for learning,

I mostly listened, and I learned so much from everyone, so much. I think there was like one or two staff doing each session but honestly it was like there were ten teachers some days. And all in my living room.

This evocative expression of having “ten teachers in my living room” illustrates how she was able to access the group's collective knowledge and experience from the comfort of her own home. It conveys a sense of safety and ease, which appeared to facilitate meaningful engagement with the core therapeutic aspects of the education programme. In addition, it highlights the powerful role of peer learning within the online group setting and how knowledge was co‐constructed in the group. Participants frequently referenced the value of hearing different perspectives and strategies, with Frank noting, “it was good to hear what other folks had tried and the advice from all the different professionals”. The group setting meant that they were not only guided by facilitators but also learned from peers whose lived experiences carried authenticity and relevance. While many participants found value in hearing from others, some participants found aspects of the group dynamic frustrating. In contrast to Lisa's positive experience of having “ten teachers” described above, Margaret found “my head was spinning listening to the different voices and opinions”, suggesting a degree of overwhelm. Other challenges were noted with Kate commenting, “Sometimes one or two people went off on one and the nurse had to rein them in”. Her language conveys frustration and the metaphor of “rein in” suggests she felt there was an element of struggle in the group experience. Participants also reflected on how the onboarding process could better support entry into the programme, with Tony conveying the emotional toll of beginning in this reflection, “It was definitely nerve‐wracking like, I didn't know what I was in for, I didn't sleep a wink the night before the first one”. Margaret echoes this experience, capturing the gap between what was provided and what was needed, “I felt so nervous about it all to begin with. If I'd had a bit more info beforehand, I think I would've felt less nervous going in”. These reflections speak to the strain of beginning the programme and the importance of mitigating the uncertainty and reducing barriers to participation.

In sum, the theme “It was a stretch” and its subthemes “Challenge and Growth” and “Ten teachers in my living room” reflect the dynamic tension between discomfort and growth. While the programme initially presented cognitive, emotional, and social challenges, these tensions laid the foundation for insight and shared understanding.

### 3.2 (2) “And Now I can Move on”

This theme captures participants' reflections on how the programme facilitated a turning point in their relationship with chronic pain and a reorientation from passive treatment seeking toward strategies grounded in contemporary pain science.

#### 3.2.1 A Breath of Fresh Air

Participants expressed a sense of relief upon realising they were not alone in their struggles with chronic pain, as articulated by Ruth

It's just a breath of fresh air that they all know what it's like. And it was really clear you know, obviously the other people with pain know what it's like but also like the nurse and the physio and stuff they seemed to understand too.

The metaphor “a breath of fresh air” conveys a movement from a sense of stagnation toward renewal and hope. The account above encapsulates the participant's experience of feeling understood, not only by peers but also by healthcare professionals intimating a sense of connection that bridges the traditional divide between patient and provider. This atmosphere of acceptance appeared to facilitate a shift in perspective and played a foundational role in her ability to move forward. Ruth used the word “fresh” repeatedly throughout her interview, describing the programme as “a fresh, fresh start” and “an opportunity to start fresh”. This repetition underscores the perceived potential for change and an orientation to a more hopeful future. The notion of “a fresh start” was held by several participants and underpinned by a recognition that life need not be on hold because of pain. Mark says,

It gave me encouragement to do things again and not just keep waiting for the pain to go away. I mean my family have been telling me this for a long time, but I don't know, I suppose it's just different when you hear it from the experts.

The credibility of the clinical team has helped facilitate a critical cognitive and emotional shift, letting go of the elusive goal of complete pain relief and instead focusing on how to live alongside pain.

#### 3.2.2 A Wider Horizon

From a foundation of acceptance and validation, participants began to reconnect with a sense of increased possibility and opportunity. Susan notes, “It feels like I've got more options now. I would say I'm, I'm probably a bit more confident in knowing about when to take painkillers and when not to, you know. There's so many other things to try”. Many spoke of making plans to retry treatments they previously felt had no benefit. James reflects on his experience with physiotherapy,

I mean yeah I've done physio before, but it didn't work but I'm going to give it a go again because you know they told me that even though it might not take the pain away there's all these benefits.

His attempt to balance past disappointment with a renewed intention to engage conveys ambivalence. The phrase “even though it might not take the pain away” suggests an emerging acceptance of the possibility of ongoing pain. Rather than expecting a cure, it seems he is beginning to reframe physiotherapy as offering alternative benefits. His willingness to persist with treatment despite uncertain outcomes also indicates growing psychological flexibility and a shift from his discouraged stance of earlier toward one of re‐engagement. Several of the participants began to engage with pain in more flexible and constructive ways as expressed by Kate.

The programme has shown me there's no easy fix and it's like, it's like all little fixes all together. So, I go for a walk now. And I meet my sister for coffee. The pain's still there mind you but life's opening up again.

This acceptance and move to engagement suggests the internalisation of some of the self‐management strategies. The feeling of life opening up is echoed across a number of accounts, vividly expressed here by Tony, “There was a stage where it felt like I was shutting down. And life was shutting down. And now I'm sort of opening up”. The use of the phrase “shutting down” conveys a period of withdrawal or disconnection. By extending this feeling to “life”, he implies that his entire world had become diminished. The contrast introduced by “now I'm sort of opening up” marks a tentative but meaningful movement toward re‐engagement. His phrasing suggests a fragile but growing sense of agency and a release from a constricted state. A recurrent thread weaving through and across participants' accounts was this sense of expansion, encompassing both an appreciation for broadened treatment possibilities and an enhanced perception of self and future. Susan concluded her interview by saying, “I don't really know how to express it. I just feel lighter somehow. I feel like I can see further”. This felt expansion signalled participants' readiness to move beyond previous constraints and engage with life in new ways.

In summary, the theme “And now I can move on”, through the subthemes “A breath of fresh air” and “A wider horizon”, captures a significant shift in participants' lived experience. From a foundation of acceptance, participants began to reconnect with hope. The programme supported this forward movement, offering not just information but an environment in which new possibilities could be imagined and pursued.

## 4 Discussion

Using IPA, this study explored how individuals experienced an interdisciplinary, group chronic pain education programme, remotely delivered via videoconferencing. Two principal themes developed: (1) “It was a stretch”, reflecting early ambivalence toward the online format and unfamiliar content, and (2) “And now I can move on”, signifying increased understanding, emotional relief, and a willingness to consider a wider range of treatment strategies. Participants described a shift from a narrow biomedical conception of pain toward a biopsychosocial understanding, and crucially, this shift was accompanied by meaningful reorientation to evidence‐based treatments and self‐management strategies.

Consistent with prior research (Louw et al. 2016; G. L. Moseley 2003; Moseley et al. 2004; Robinson et al. 2016), our findings suggest that participation in the education programme supported a process of pain reconceptualisation. This cognitive shift involves moving from understanding pain as a direct indicator of tissue damage to recognising it as a complex, multifactorial experience influenced by biological, psychological, and social factors (G. L. Moseley 2007). Participants in the current study described initially holding strong beliefs in biomedical causes and treatments for pain, and some resistance to having these views challenged, a finding frequently reported in the literature (Dong and Bäckryd 2023). Such assumptions, while understandable, are associated with increased distress, higher healthcare utilisation, and disengagement from self‐management behaviours (Buchbinder et al. 2018; Jensen et al. 1999; Morton et al. 2019; Walsh and Radcliffe 2002). What emerged through the programme was a gradual re‐evaluation of these beliefs, facilitated by accessible explanations of modern pain science and the opportunity to reflect on personal experiences in a supportive group setting.

Our study extends the work of Robinson et al. (2016), whose qualitative investigations into pain neurophysiology education offered foundational insights into the processes and challenges of reconceptualization and pain education more broadly. Their initial single‐session, in‐person intervention showed partial reconceptualization, with perceived relevance and personal applicability shaping outcomes. A subsequent pre‐post interview study highlighted how pre‐existing beliefs could both enable and constrain reconceptualization, and how the degree of conceptual shift could influence the clinical impact of education (King et al. 2016).

We build upon this work by examining a longer‐format, interdisciplinary programme delivered remotely via videoconferencing. This format enabled a more sustained and relational approach to education and improved accessibility. Participants described shifts not only in their understanding of pain but also in their expectations of what effective treatment could involve. This shift in expectations is clinically significant, as discrepancies between expectations and the realities of chronic pain management can negatively affect satisfaction and quality of life (Geurts et al. 2017). By supporting participants to revise their expectations in line with contemporary pain science, the programme appeared to reduce this dissonance, fostering greater acceptance and engagement. Participants expressed increased openness to physiotherapy‐led interventions and to self‐management strategies like pacing, approaches they had previously viewed as secondary or inadequate. This reorientation is particularly important because, while many effective treatments for chronic pain exist (Cohen et al. 2021; Odling‐Smee 2023; Vowles et al. 2020), their impact is often limited by patients' difficulty in understanding the value of engagement (Ryan et al. 2024). Helping participants view these approaches as legitimate and meaningful, rather than inferior to biomedical interventions, may therefore enhance both uptake and long‐term adherence to evidence‐based treatments.

Participant accounts suggest that this reorientation may have been facilitated by specific features of the current education programme. In particular, the group‐based, interdisciplinary, and multi‐session structure appeared to provide the relational and temporal scaffolding necessary to support more than just conceptual shifts and enable a deeper integration of new treatment perspectives (Dong and Bäckryd 2023). Participants described the opportunity to hear about the lived experiences of others as particularly impactful, helping to legitimise their own struggles and alleviate feelings of marginalisation, elements which are important given the isolating nature of chronic pain (Noguchi et al. 2024). While peer support has long been recognised as a key component of interdisciplinary pain management programmes (Farr et al. 2021), its specific role within pain education interventions has received comparatively less empirical attention.

Participants also valued the diversity of clinician perspectives available to them (Nicosia et al. 2021). These observations reinforce emerging evidence that group cohesion and therapeutic alliance can be cultivated effectively in online formats provided facilitation is intentional and well structured (Buhrman et al. 2016). Furthermore, the remote delivery of the programme emerged as a key enabler of access. Being able to engage from home supported sustained attendance, allowing individuals to benefit from the cumulative, relational aspects of the programme that might otherwise have been inaccessible in traditional settings (Booth et al. 2022). Crucially, the extended format enabled individuals to revisit, question, and gradually incorporate unfamiliar ideas over time. This contrasts with shorter‐duration education models, which may prompt initial cognitive reframing (Robinson et al. 2016) but lack the continuity required for orientation toward meaningful change.

These findings should be considered within the context of certain limitations. While IPA provides idiographic insight into lived experience, it does not aim for generalisability. The sample size, appropriate for IPA, limits the diversity of perspectives, and data were drawn from a single programme site, which may not reflect experiences elsewhere. The self‐selecting sample may have included individuals more comfortable with online formats and psychosocial approaches. The perspectives of those who declined participation or dropped out were not captured, potentially biasing the findings toward more positive experiences, particularly among those who did not encounter digital barriers or misaligned expectations. Finally, data were collected at a single time point; a longitudinal design would be valuable in future research to explore the durability of reconceptualization and treatment reorientation.

The experiential accounts reported here, while predominantly positive by the later stages of the programme, were not uniformly so throughout participants' trajectories and should be interpreted in light of the broader evidence base in which online group‐based pain management interventions demonstrate mixed clinical outcomes and more heterogenous participant experiences (Zarnegar and Booth 2025). The discrepancy between favourable subjective experiences and more variable outcome data may reflect the distinction between felt therapeutic gains, such as feeling validated and gaining conceptual clarity, and measurable changes in pain intensity or function, which may take longer to emerge or depend on factors beyond programmes. In addition, Pain Retrained was specifically an education programme rather than a comprehensive interdisciplinary programme focused on behaviour change (e.g., Gatchel et al. 2014). Previous research has indicated that educational and psychological components tend to be well received in remote formats, whereas physical rehabilitation elements are often more challenging to deliver effectively online (Willcocks et al. 2023). As noted above, it is also possible that individuals who had more negative experiences were underrepresented in our sample, reinforcing the need to explore potential barriers such as digital literacy in future work.

Although the qualitative design and small sample limit generalisability, the insights gained offer a strong foundation for shaping future education‐based interventions for people with chronic pain. We recommend that future programmes prioritise accessibility, peer connection, synchronous interdisciplinary facilitation, and multi‐session formats. Improved onboarding procedures may help mitigate early uncertainty and facilitate stronger initial engagement. Our data also suggest that incorporating lived‐experience perspectives early in the programme, through peer facilitators or structured narrative‐sharing opportunities, may enhance perceived legitimacy, foster shared identity, and promote hope (Mathias et al. 2014). While online formats offer opportunities for scalability, maintaining fidelity to these key elements will be important as programmes expand.

Our findings also highlight the importance of qualitative research in capturing patient perspectives on pain education and online interventions. While quantitative approaches remain important for assessing pain‐related outcomes (Leake et al. 2021), qualitative inquiry offers unique insights into the processes and meanings underlying those changes (Kazdin 2007). In this study, participants revealed key psychological shifts, most notably a reorientation to treatment following reconceptualization of pain. This reorientation reflects a central purpose of pain education: not merely to transfer knowledge but to foster readiness for change and promote meaningful engagement with evidence‐based care. These insights carry significant implications. If such reorientations are not captured, educational interventions such as this one risk being dismissed as ineffective and consequently undervalued and underfunded, an issue of increasing relevance given the rising demand for accessible, scalable approaches within pain services (Souza et al. 2025). Existing quantitative measures, such as the Pain Stages of Change Questionnaire (Kerns and Rosenberg 2000), may reflect related domains but do not sufficiently capture the layered cognitive and affective shifts identified here. There is therefore an argument for developing a more sensitive, multidimensional outcome measure that reflects the processual nature of pain reconceptualization and treatment reorientation.

In conclusion, this study offers previously unexplored detail about the experiences of individuals participating in an interdisciplinary, group‐based pain education programme delivered via videoconferencing. It builds on earlier findings about the role of education in the reconceptualization of chronic pain and adds to current knowledge by highlighting how an educational intervention can incorporate relational and temporal elements which facilitate participant reorientation to evidence‐based treatments. Integrating the views of people with lived experience is important in the development and evaluation of interventions so that they may better address the complex needs of those living with chronic pain.

## Author Contributions

M.M. designed the study, collected and analysed the data, and drafted the manuscript. M.D. and K.E.V. contributed to the interpretation of the results. All authors critically evaluated the findings and edited the manuscript. All authors have approved the final version of the manuscript.

## Funding

This research was conducted with the support of PhD funding from the Northern Ireland Department for the Economy.

## Conflicts of Interest

The authors declare no conflicts of interest.

## Supporting information


**Appendix S1:** Outline of pain retrained programme.

**Appendix S2:** Interview schedule.

**Table S1:** Participant characteristics.
