# Supplementary material for: Pain Retrained: Participant Perspectives of an Online, Interdisciplinary Chronic Pain Education Programme
Source: Eur J Pain. 2026 Jan 29;30(2):e70223. doi: 10.1002/ejp.70223 (PMC12854195; doi:10.1002/ejp.70223)
Supplement: Supplementary file 1 — Appendix S1: Outline of pain retrained programme. [file EJP-30-0-s002.docx]

**Appendix S1- Outline of Pain Retrained programme**

The Pain Retrained programme is a group-based pain education intervention underpinned by biopsychosocial and cognitive-behavioural understandings of chronic pain. It serves as the primary entry point for most individuals referred to the specialist pain service, offering access to evidence-based education and engagement with the interdisciplinary team. It provides patients with core knowledge and conceptual tools for making sense of their pain experiences.

The intervention consists of six weekly sessions, each two hours in length, delivered remotely via Microsoft Teams. Groups typically include 15 to 20 participants and are jointly led by two clinicians. Members of the interdisciplinary team contribute to delivery, including a clinical psychologist, physician, occupational therapist, physiotherapist, and specialist pain nurses. Each session combines structured teaching with interactive elements: clinicians present material aligned with the weekly topic, followed by guided group discussions that support consolidation of concepts and promote reflection. Engagement is facilitated through live conversation and the chat feature, with participants encouraged to share their experiences and viewpoints.

Session topics cover an introduction to the biopsychosocial perspective on pain, highlighting cognitive, emotional, and behavioural influences. In addition, there is discussion of the role of movement and physical activity, approaches to sleep, stress, and flare management, principles of medication use and potential risks, and an overview of the evidence supporting common treatment strategies. All participants receive a workbook containing session slides, summaries of key messages, reflective tasks, and links to additional reputable resources. The programme follows a standardised format rather than being individually customised, although facilitators may adjust emphasis or pacing in response to group needs. Facilitators are trained to deliver the content and follow a structured manual. Fidelity is maintained through co-facilitation and weekly interdisciplinary meetings in which the team reflects on session delivery and addresses implementation issues. Although fidelity was not formally scored, these processes supported consistency, and no changes to the programme were introduced during the study period.
