# Supplementary material for: Pain Retrained: Participant Perspectives of an Online, Interdisciplinary Chronic Pain Education Programme
Source: Eur J Pain. 2026 Jan 29;30(2):e70223. doi: 10.1002/ejp.70223 (PMC12854195; doi:10.1002/ejp.70223)
Supplement: Supplementary file 2 — Appendix S2: Interview schedule. [file EJP-30-0-s001.docx]

**Appendix S2 - Interview Schedule**

**Introduction:**

Hello, my name is Maura McCarron and I am a PhD candidate in the School of Psychology at Queen’s University Belfast. It is lovely to meet you and thank you for taking part in this study. As detailed within the participant information sheet, I am here to interview you as part of a project which explores the experiences of people who have taken part in the Pain Retrained programme.

This interview should last approximately forty-five minutes. When I begin asking you the interview questions, I will start recording. Are you happy to continue taking part in this

interview? (If yes, continue with script)

Before we begin with questions, I would like to confirm some of your details. I am

going to write this information down, however this will be kept separately from your

interview recording. Are you happy to continue? (If yes, ask demographic questions

and record these on proforma).

Do you have any questions before we start? If you are happy to proceed, we will

begin the interview and I will start recording now.

**Interview:**

1. Please tell me what it was like for you taking part in the Pain Retrained programme?
2. What were your expectations of the programme? Did you have any concerns before starting?
3. What were your first impressions? Did these change as the programme progressed?
4. Have you ever done anything like Pain Retrained before? Give examples/how did this compare?
5. What were the positive aspects of the programme?

Example of best experience(s)

1. What were the negative aspects of the programme? Examples of the worst experience(s)
2. What changes have you noticed since taking part in the programme?

(e.g., how you feel about yourself? Any change in your mood/ability to do activities?

Are these changes big/small? Have your family/friends noticed any changes?)

1. Do you think any changes were as a direct result of taking part in the Pain Retrain programme?

Any other factors responsible?

1. Have you incorporated any of the advice into your daily life?

What in particular? Has this been helpful?

1. Do you view yourself any differently since completing the programme?
2. Would you recommend this programme?

Would you suggest any changes?

1. What are your plans for managing your symptoms from this point?

**Interview closure:**

That is the end of my questions, thank you for taking part. Is there anything else that

you would like to add? Perhaps there is something I missed, or something that we

have not considered in enough detail?

As a reminder, you can withdraw from this study up until one week after today. After that, your data will be made anonymous so you will be no longer able to withdraw. If you decide you would like to withdraw between now and then, please contact myself or my

supervisors through our contact details which are included within the study information sheet. Thank you again for participating in this study, your involvement is very much appreciated.
