# Supplementary material for: Pain Retrained: Participant Perspectives of an Online, Interdisciplinary Chronic Pain Education Programme
Source: Eur J Pain. 2026 Jan 29;30(2):e70223. doi: 10.1002/ejp.70223 (PMC12854195; doi:10.1002/ejp.70223)
Supplement: Supplementary file 3 — Table S1: Participant characteristics. [file EJP-30-0-s003.docx]

Table S1. Participant characteristics

| Pseudonym | Age (years) | Gender | Employment status | Pain Diagnosis | Pain Duration (years) |
| --- | --- | --- | --- | --- | --- |
| Anne | 65 | Female | Employed | Low back & pelvic pain | 5 |
| Frank | 68 | Male | Retired | Back pain & Fibromyalgia | 4 |
| James | 55 | Male | Employed | Post-injury | 25 |
| Kate | 45 | Female | Employed | Post-injury | 6 |
| Lisa | 52 | Female | Carer for grandchildren | Chronic headache | 5 |
| Margaret | 67 | Female | Retired | Osteoarthritis & Fibromyalgia | 8 |
| Mark | 42 | Male | Employed | Post-injury | 19 |
| Ruth | 50 | Female | Not in paid work | Fibromyalgia | 16 |
| Susan | 59 | Female | Carer for grandchildren | Fibromyalgia | 10 |
| Tony | 64 | Male | Employed | Post-injury | 20 |
